# Scale‐up of a monoclonal antibody CHO fed‐batch production in stirred tank bioreactors: Effect of hydrodynamic conditions and feeding regimen

**DOI:** 10.1002/btpr.70073

**Published:** 2025-09-29

**Authors:** Lucas Lemire, Sebastian‐Juan Reyes, Yves Durocher, Robert Voyer, Olivier Henry, Phuong Lan Pham

**Affiliations:** ^1^ Department of Chemical Engineering Polytechnique Montreal Montreal Quebec Canada; ^2^ Human Health Therapeutics Research Centre National Research Council Canada Montréal Quebec Canada

**Keywords:** aeration strategy, bioreactor production, CHO fed‐batch, feeding regimen, monoclonal antibody, scale‐up

## Abstract

Key hydrodynamic‐related parameters such as volumetric power input (P/V), impeller configuration, aeration strategy, and maximum gas sparge rate, as well as an appropriate feeding strategy, must be carefully selected to improve production yields in bioreactor. In this study, the feeding regimen was found to have an important impact on cell growth and productivity of a cumate‐inducible CHO fed‐batch cell culture. A low‐volume feeding regimen avoided a rapid increase in osmolality, allowing for prolonged cell viability and a 33% increase in volumetric titer compared to the high‐volume feeding regimen. Both sparged air and oxygen were used for dissolved oxygen (DO) control, utilizing three levels of airflow rates. An optimum airflow rate of 0.0031 vvm was found to improve cell growth, longevity, and thus final titer. A larger air cap required increased gas flow rates, which led to an earlier cell mortality. Scale‐up from 1‐L to 10‐L bioreactor using constant P/V and air cap volumetric gas flow rate (vvm) allowed for comparable cell growth and productivity. Further investigation of the effect of mixing and aeration was done by maintaining P/V and vvm constant throughout the cell culture, which further improved product titers at 11 days after induction. Our study also demonstrates that keeping a constant volume by removing a culture amount equal to the feed volume added at each sampling event can significantly improve the final volumetric titer. This finding shows the benefit of developing a concentrated feed to reduce the volume increase, which in turn could greatly ease the scale‐up task.

## 1 INTRODUCTION

Chinese Hamster Ovary (CHO) cells are the established workhorse for producing recombinant therapeutic proteins such as monoclonal antibodies (mAb) due to their ability to produce human‐like proteins. In order to satisfy the ever‐increasing demand for these biologics, there have been major improvements in cell engineering,^1^, ^2^ media composition,^3^ and cellular microenvironments such as shear stress,^4^ pH levels,^5^ and carbon dioxide levels.^6^, ^7^ However, the scale‐up of these cell cultures in bioreactors, which is necessary to supply market needs, remains a nontrivial task. Bioreactor scale‐up challenges are due to differences in shear stress levels, gas transfer capabilities, and the formation of undesired concentration gradients (e.g., nutrients, gasses, and pH) in larger vessels. Operating parameters in bioreactor design can be divided into scale‐independent (e.g., temperature, pH, dissolved oxygen, and feed regimens) and scale‐dependent variables (e.g., impeller tip speed, mass transfer coefficient k_L_a, gas residence time, and mixing time).^8^ While scale‐independent parameters can generally be matched across scales, scale‐dependent variables are interdependent, making it impossible to maintain all of them constant during scale‐up. For instance, maintaining constant mixing times across scales increases impeller tip speeds, potentially damaging cells. Alternatively, maintaining impeller tip speeds constant reduces agitation, potentially leading to poor mixing. Similarly, as bioreactors increase in size, the reduced surface area‐to‐volume ratio impacts surface gas transfer, necessitating higher sparged gas flow rates, which may also cause cell damage due to bubble shear stress. Many different strategies, most notably constant mass transfer coefficient, constant volumetric power input, and constant volumetric gas flow rate, have been applied for scaling up of CHO cell culture processes. However, given the trade‐offs mentioned, there is no universally applicable method for bioreactor scale‐up such that the optimal scale‐up approach is dependent on the needs of the process.^8^ As the most prevalent scale‐up criteria (constant kLa, P/V, impeller tip speed, and gas flow rates) pertain to mixing and gassing, it is important to optimize these parameters in small‐scale bioreactor vessels before large‐scale production.

In fed‐batch, which is the most prevalent mode of cell culture due to its simplicity and efficacy, a highly concentrated nutrient solution is added throughout the cell culture. An unoptimized feeding strategy may result in a lack of nutrients required for cell growth and protein production, an accumulation of intermediate metabolites, and a sharp rise in osmolality. Glucose depletion (below 2 mM) has been linked to a drastic reduction of intracellular ATP and certain intracellular amino acids,^9^ which in turns leads to a decrease in specific productivity and deficient glycosylation of the recombinant proteins.^10^ Asparagine and aspartic acid depletions have been linked to an arrest in cell growth and antibody production, which only restarts after these nutrients are added back to the media.^11^, ^12^ Alanine was found to negatively affect biomass production by serving as a signal indicating that TCA cycle intermediates, such as α‐ketoglutarate,^13^, ^14^ are in abundance.^15^ On the other hand, accumulation of certain amino acids (glycine, leucine, methionine, phenylalanine, serine, tryptophan, and tyrosine) is known to inhibit cell growth.^16^ Higher ammonia accumulation has also been observed in high‐volume feeding regimens^17^ which is known to negatively impact growth, production, and glycosylation of the recombinant protein.^18^ When a concentrated feed is added to the cell culture, this creates a spike in osmolality. Osmolality levels of 460–500 mOsm/kg have been shown to reduce viable cell density and viability.^19^

Mixing of CHO cell culture across all scales is primarily done through an axial flow impeller such as one with pitched‐blades.^8^ To optimize cell culture mixing, the main parameters that can be changed are: the mixing speed, the number of impellers, the liquid fill volume, and the pumping direction of the impeller. Up‐pumping impellers lose less power while down‐pumping impellers have flow instabilities as well as torque and power fluctuations such that up‐pumping impellers are generally favored.^20^ A reduction of the fill volume increases P/V, allowing for better mixing at the same impeller tip speed. Although tip speed is maintained, the volumetric power applied to the liquid is greater; this increases energy dissipation rates and in turn shear stress applied to cells. Furthermore, low fill volumes lead to an improvement in oxygen transfer rates.^21^ However, in order to maximize production and minimize the production footprint, fill volumes are typically increased aiming to achieve near 100% maximum operating volume at harvest. The mixing speed is important as the movement of the impeller can be a source of shear stress on mammalian cells which are sensitive to such forces compared to microbial fermentation. To avoid cell damage, the impeller tip speed is typically kept below 1 m/s in benchtop bioreactors.^8^ Furthermore, mixing speed is used to estimate the volumetric power input (P/V) of the culture broth. The P/V is a measure of how much power is applied to the liquid during mixing and as such is used to ensure proper homogenization of media. This parameter is typically kept between 10 and 80 W/m^3^.^8^ However, in large‐scale single‐use bioreactors the maximum P/V is typically between 20 and 30 W/m^3^ due to physical limitations.^22^ As for the number of impellers, it impacts gas transfer and the shear stress applied to cells. The P/V of double impeller bioreactors can be estimated by doubling the power number (Np) of a single impeller.^23^ When multiple impellers are used and the same P/V is maintained by adjusting the mixing speed, the gas transfer coefficient, k_L_a, has been shown to be lower in double impeller configurations than that of single impeller systems.^24^ This is captured in the empirical models, as the estimation of P/V is calculated using a power number (Np) which is changed when multiple impellers are added. The use of a double impeller spreads the mixing force across a larger volume changing the local k_L_a distribution. A higher k_L_a in single impeller systems indicates that focusing the mixing power near the site of bubble formation improves gas transfer.^24^ On the other hand, a double impeller system allows for a lower mixing speed, and thus impeller tip speed, at the same P/V as a single impeller system, which is desirable in reducing shear stress applied to cells.

In this study, we use cumate‐inducible CHO cell clones to explore the impact of feeding volume and hydrodynamic conditions on recombinant monoclonal antibody production. The cumate gene switch works by constitutively expressing the cymene repressor (CymR), which binds to the operator sites (CuO). In the absence of cumate, the binding of CymR to CuO prevents the expression of the cumate reverse transactivator (rcTA) that is under the control of the CMV5‐CuO promoter. When added, cumate binds to CymR and causes a conformational change resulting in its disassociation from the CuO site and allowing rcTA to bind to the CR5 promoter, hence driving the expression of the transgene of interest.^25^ This allows us to separate the cell culture into two distinct phases: cell growth and protein production. The separation of the process into two phases results in a higher productivity by allowing for rapid cell growth before induction and boosting protein production at high cell density after induction.^26^ Process optimization and scale‐up to 10‐L were done using the cell clone producing product A. First, feed regimens were explored, and their impacts on osmolality and product titer were investigated. Second, the number of impellers, along with different aeration strategies, is studied. The differences in physicochemical environments caused by the number of impellers have been extensively studied in our work, while in the literature, few studies present the effect of these specific configurations on cell culture.^27^ Positioning of impellers and the resulting flow patterns could potentially have impacts on cell cultures; however, this is out of scope for this study. As for gassing, an aeration cascade is used where air is sparged for dissolved oxygen (DO) control until a specified air flow rate (further referred to as “air cap”) is reached, after which the air flow rate is supplemented with pure oxygen as needed. The process is scaled up from 1‐L to 10‐L by using concurrently the constant P/V and constant gassing as scale‐up criteria. The developed 1‐L process is also transferred to two different clones of a different mAb (product B) to demonstrate its robustness. The process utilizing the clone production of product B is used to further explore the impact of mixing and gassing microenvironments on cell culture productivity. The main novel contributions of this study are the characterization of a lesser‐known gassing strategy, referred to as cascade aeration, and the exploration of the effect of air cap amplitude on cell culture. Additionally, investigating different methods of maintaining a constant P/V and vvm throughout cell cultures provides insights into understanding how the hydrodynamic conditions can impact culture performance.

## 2 MATERIALS AND METHODS

### 2.1 Small‐scale cell culture maintenance and pre‐culture

Two different inducible CHO‐GS stable clones with a cumate gene switch producing distinct monoclonal antibodies Palivizumab (PLVZM, hereinafter referred to as Product A) and Omalizumab (OMLZM, hereinafter called Product B) were used. The proprietary cell lines were generated internally as previously reported.^26^ The CHO^BRI/55E1^ cell line producing Product A (PLVZM) differs slightly from the CHO^BRI2353^ cell line expressing Product B (OMLZM). Both cell lines come from the same parental CHO^BRI^ cell line, which is derived from the DHFR deficient CHO‐DXB11.^28^ The CHO^BRI^ cell line has been adapted to suspension growth in serum‐free culture media. The CHO^BRI/55E1^ parental cell line is equipped with the cumate (p‐isopropylbenzoate) inducible expression system as it stably expresses the cymene repressor (CymR) and the cumate reverse transactivator (rcTA). Thus, clones will have high recombinant protein expression in the presence of cumate and low basal expression in the absence of cumate.^26^ The CHO^BRI2353^ parental cell line is equipped with rcTA only. For constitutive expression, the parental cells are simply transfected with the plasmid DNA containing the gene of interest (GOI) under the control of the cumate promoter (CR5). For inducible expression, cells are transfected with a plasmid DNA containing the GOI along with a second expression cassette for constitutive expression of the CymR repressor. In this later case, cumate addition is required to induce expression of the GOI.^29^

Cells were stored frozen in BalanCD CHO Growth A medium (Fujifilm Irvine Scientific, USA) with 7.5% (v/v) dimethylsulfoxide (DMSO) (Sigma Aldrich, ≥99.7% purity, USA) in liquid nitrogen storage tanks (MVE, Series 800–190, USA) at −180°C. The cells were thawed using a water bath (Fisher Scientific, Isotemp Digital 2320, USA) at 37°C for 5 min. Cells were expanded in shake flasks (Corning, USA) ranging from 125 mL to 2 L in incubators regulating temperature at 37°C, humidity at 75% RH, and mixing at 120 RPM with a 25 mm orbit. Cells were passaged every 2–3 days in BalanCD CHO Growth A medium supplemented with 50 μM L‐methionine sulfoximine (MSX) (Sigma‐Aldrich, USA) to maintain cell density between 0.2 and 3.0 million cells/mL.

### 2.2 Bioreactor cell culture and operating conditions

Five experimental conditions (Table 1) were developed to study the effect of bolus feeding volume, aeration, and mixing strategy on cell growth and recombinant protein production. Cell cultures were performed in 1.3‐L and 14‐L BioFlo120 (Eppendorf, Germany), with maximum working volumes of 1 and 10 L, respectively, under fed‐batch mode. The bioreactors were inoculated at 0.3 million cells/mL. BalanCD CHO Growth A medium was supplemented with 0.1% (w/v) Kolliphor P188 (BASF, Germany) for a total surfactant concentration of 0.2% (w/v) and 50 μM MSX (Sigma‐Aldrich, USA). Cells were fed with 0.8X BalanCD CHO Feed4 (Fujifilm Irvine Scientific, USA) in a bolus mode on the induction day and fed every two or three days onward. At induction, a bolus of 0.1% (v/v) of FoamAway™ Antifoam (Thermo Fisher Scientific, USA) is added with additional supplementation of 0.1% (v/v) bolus as needed to avoid excessive foaming at the surface.

**TABLE 1:** Bioreactor operating conditions.

Parameter	Experimental conditions					
	Low feeding strategy	High feeding strategy	1‐Impeller/Medium air cap	Low air cap	High air cap	Scale‐Up
Production Vessel	1.3 L BioFlo120	1.3 L BioFlo120	1.3 L BioFlo120	1.3 L BioFlo120	1.3 L BioFlo120	14 L BioFlo120
Initial Working Volume (mL)	800	800	650	650	650	7000
Feed Regimen	Low Feeding Strategy	High Feeding Strategy	Low Feeding Strategy	Low Feeding Strategy	Low Feeding Strategy	Low Feeding Strategy
Number of Impellers	2	2	1	1	1	1
Impeller Diameter (m)	0.058	0.058	0.058	0.058	0.058	0.1
Agitation Rate (RPM)	148	148	171	148	147–174	130–160
Initial P/V (W/m^3^)	35	35	35	23	22 from −3 to 2 DPI	22 from −3 to 2 DPI
Final P/V (W/m^3^)	27	27	27	18	29 from 2 DPI	29 from 2 DPI
Surface Air Flow Rate (mL/min)	25	25	25	25	21.5–27	230–330
Initial Surface vvm	0.031	0.031	0.038	0.038	0.033	0.033
Final Surface vvm	0.024	0.024	0.024	0.024	0.033	0.033
Sparging Air Cap (mL/min)	2	2	2	1	2–12.3	22–150
Sparging Air Cap vvm	0.0025	0.0025	0.0031	0.0015	0.0031 from −3 to 0 DPI, then 0.015	0.0031 from −3 to 0 DPI, then 0.015

The inducible cell system, previously described by our research team,^25^ allows for two distinct phases during the cell culture: growth and production. The growth phase lasts from −3 to 0 days post induction (DPI). At the end of the growth phase, the cultures were induced with 2 μg/mL of cumate (4‐Isopropylbenzoic acid (Ark Pharm Inc., USA)). Throughout the growth phase and the beginning of the production phase, from −3 to 2 days post induction (DPI), temperature was maintained at 37°C, after which it was decreased to 32°C for the remainder of the production phase. pH was maintained at 7.0 ± 0.2 by carbon dioxide sparging and addition of 4% (w/v) sodium hydroxide and 9% (w/v) bicarbonate base solution to the culture. The 1‐L benchtop bioreactors were agitated with a 5.8 cm diameter 3‐blade 45° pitched‐blade impeller at a mixing speed setpoint of 148 RPM, such that the volumetric power input was estimated at 35 and 27 W/m^3^ for initial and final volumes respectively. The dissolved oxygen (DO) setpoint was 60% of air saturation. The DO control was achieved through constant air surface aeration of 25 mL/min (0.031 vvm, volume of gas per volume of liquid per minute) and cascade sparging aeration, which will be described in the following paragraph (Section 2.2.3). Cell cultures were terminated when cell viability reached below 70%.

#### 2.2.1 Low‐ and high‐volume feeding regimens

1‐L bioreactor cell cultures were operated at an initial volume of 800 mL (80% of the maximum fill volume). Cell cultures were fed according to low‐ or high‐volume feeding strategies (Table 2) on 2, 4, 7, and 9 DPI. In the low‐volume feeding strategy (namely low feeding, LFS), 2 M D‐(+)‐glucose (≥99.5% purity, Sigma Aldrich, USA) is supplemented as needed to maintain glucose concentration above 17 mM, while in the high‐volume feeding strategy (HFS) the culture is supplemented to bring the glucose concentration to 33 mM after feeding.

**TABLE 2:** Low‐ and high‐volume bolus BalanCD CHO Feed4 addition regimens described as a percentage of initial culture volume.

Days post induction (DPI)	Low feeding strategy (LFS)	High feeding strategy (HFS)
0	5%	5%
2	7.5%	7.5%
4	7.5%	12%
7	5%	7%
9	5%	7%
11	7.5%	7.5%
Total	**37.5%**	**46%**

#### 2.2.2 Number of impellers and mixing

**FIGURE 1:**
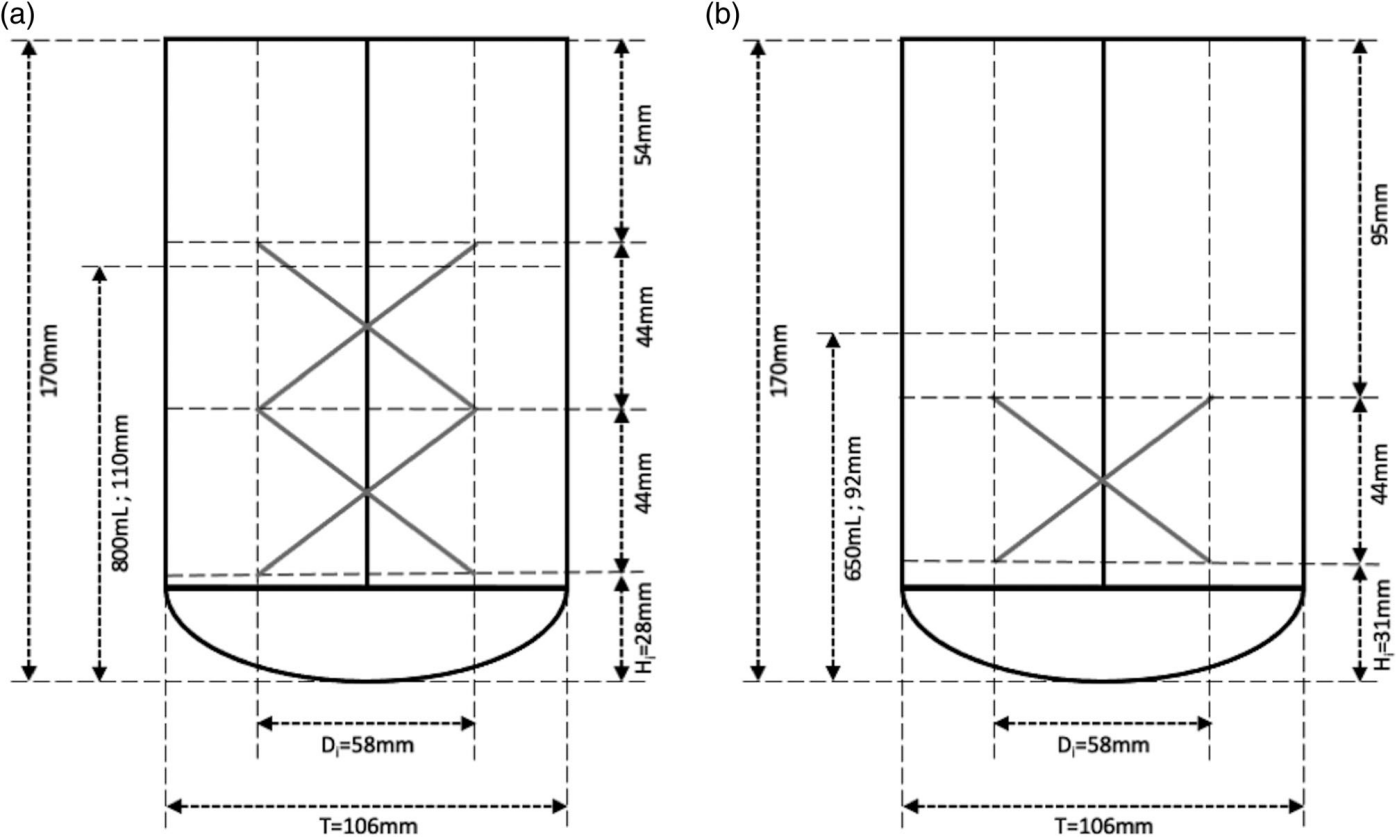
Measurements and mixing configuration of the (a) 2‐impeller and (b) 1‐impeller bioreactor. 800 and 650 mL are the initial working volumes of the 2‐ and 1‐impeller configurations respectively.

Both the 1‐ and 2‐impeller fed‐batch cell cultures were fed following the low feeding strategy (LFS). The initial volume of the 2‐impeller configuration was increased to 80% maximum fill volume when compared to the 65% maximum fill volume of the 1‐impeller configuration to assure all impellers were submerged for the entirety of the cell culture. The height of the lower impeller on the 2‐impeller configuration (H_i_) is as low as possible without spatial hindrance of the probes, gas spargers, samplers, and harvest lines (Figure 1). The second impeller is placed immediately above the first one. As both impellers are on the same shaft and rotating in the same direction, the impellers have no way to impact with each other as it might appear in Figure 1. The estimated initial volumetric power input (P/V, W/m^3^) (Equation 1) was maintained for both conditions at 35 W/m^3^ by adjusting the impeller mixing speed (N).

(1) $$P/V=\frac{N_{p}d_{i}^{5}N^{3}}{V}$$

where *N*_p_ is the impeller power number, *d*_*i*_ (m) is the impeller diameter, *N* (s^−1^) is the impeller mixing speed, and V (m^3^) is the liquid volume. The power number of 2 impellers is equal to $2\times N_{p_{1}}$ where $N_{p_{1}}$ is the power number of 1 impeller.^23^ Adjusting the mixing speed to maintain the P/V constant for both conditions leads to different impeller tip speeds ($v_{t}$, m/s) (Equation 2) of 0.45 and 0.52 m/s for the 2‐ and 1‐impeller configuration, respectively.

(2) $$v_{t}=\pi d_{i}N$$

#### 2.2.3 Aeration strategy and air cap

Dissolved oxygen control is done by cascade sparging aeration such that air is first sparged for oxygen control until reaching the air cap flowrate (AC), after which sparging is supplemented with pure oxygen as needed. The air caps of 1, 2, and up to 12.3 mL/min correspond to 0.0015, 0.003, and 0.015 volumetric gas flowrate (vvm) when initial volumes of 650 mL are used. The high air cap condition uses a constant vvm of 0.015 after induction, which results in a maximum air cap of 12.3. This flowrate was used for the high AC condition to ensure culture conditions would have observable differences. These will be referred to as low, mid, and high AC, respectively. All air cap conditions used the 1‐impeller configuration and the LFS. However, the 1 mL/min treatment used a reduced agitation rate such that the initial P/V was estimated at 23 W/m^3^. This was done to determine if a lower shear stress environment would allow maintaining cell viability longer when compared to the 2 mL/min AC treatment. The total cumulative oxygen sparged (TCO, [mL]) was calculated using Equation 3, where F is the gas flowrate (mL/min), *Δ*t is the sampling rate (min), and t_start_ and t_end_ are the start and end times of the cell culture (min).

(3) $$\mathrm{TCO}=\sum_{t_{\text{start}}}^{t_{\mathrm{end}}}\left(0.21\times F_{\mathrm{air}}\times \Delta t+F_{O_{2}}\times \Delta t\right)$$

#### 2.2.4 Scale‐up to 10‐L bioreactor

Scale‐up to 10‐L, maximum working volume, bioreactor was done by maintaining the number of impellers, the volumetric power input (P/V), the feeding strategy, and the air cap vvm constant. The volumetric power was kept constant at 22 W/m^3^ from −3 to 2 DPI and 29 W/m^3^ afterwards by adjusting the mixing speed. The AC vvm was maintained constant at 0.0033 min^−1^ by adjusting the air flowrate as volume increases. These air caps and mixing speeds were selected to ensure the maintenance of the DO set‐point in the 10‐L unit, given the oxygen sparge flowrate limitations of the experimental setup. The surface air flowrate was increased with feed additions throughout the cell culture to maintain a constant vvm of 0.033 min^−1^ for both bioreactor scales.

### 2.3 Sampling and Handling of Samples

At −3, 0, 2, 4, 7, 9, and 11 DPI, 5 mL of culture was purged before sampling another 5 mL. Then, 1 mL of sample was used for cell counts and viability by means of trypan blue exclusion staining using a Cedex MS20C Automated Cell Counter (Innovatis, Germany). Cell counts were observed to have an average relative standard deviation error of 7%. The integral viable cell concentration (IVCC, cell day/mL) and doubling time (td, h) were calculated with Equations 4 and 5 respectively, where VCD is the viable cell density, ΔIVCC (cell.day/mL) refers to the change in integral viable cell density, V (mL) refers to the current culture volume, and t (day) is the sampling time. Subscripts 1 and 2 refer to the previous and the current sampling times respectively:

(4) $${\text{IVCC}}_{2}={\text{IVCC}}_{1}+\Delta \text{IVCC}={\text{IVCC}}_{1}+\frac{\left({\mathrm{VCD}}_{2}\times V_{2}\right)+\left({\mathrm{VCD}}_{1}\times V_{1}\right)}{2}*\frac{\left(t_{2}-t_{1}\right)}{V_{2}}$$

(5) $$t_{d}=\frac{\ln\left(2\right)}{\mu }=\frac{\ln\left(2\right)}{\ln\left({\mathrm{VCD}}_{2}/{\mathrm{VCD}}_{1}\right)/24\left(t_{2}-t_{1}\right)}$$

The remainder of the sample was centrifuged at 5000× g for 5 min to obtain the supernatants. Glucose, lactate, and ammonia concentrations of sample supernatants were determined by colorimetric assays using Vitros350 (Ortho‐Clinical Diagnostics, USA). Culture media supernatant osmolality was measured by OsmoTECH osmometer (Advanced Instruments, USA). Cell specific consumption rates of glucose (qGlu, pmol/cell/day) and production of lactate (qLac, pmol/cell/day) and ammonia (qNH_3_, pmol/cell/day) were calculated using (Equations (6), (7), (8)), respectively, where [Glu] (pmol/mL), [Lac] (pmol/mL), and [NH_3_] (pmol/mL) refer to the concentrations of glucose, lactate, and ammonia; subscripts 1 and 2 refer to the previous sampling time (after feeding) and the current sampling time (before feeding), respectively:

(6) $$\text{qGlu}=\frac{{\left[\mathrm{Glu}\right]}_{1}-{\left[\mathrm{Glu}\right]}_{2}}{∆\text{IVCC}}$$

(7) $$\text{qLac}=\frac{{\left[\mathrm{Lac}\right]}_{2}-{\left[\mathrm{Lac}\right]}_{1}}{∆\text{IVCC}}$$

(8) $$\mathrm{qN}H_{3}=\frac{{\left[{\mathrm{NH}}_{3}\right]}_{2}-{\left[{\mathrm{NH}}_{3}\right]}_{1}}{∆\text{IVCC}}$$

The resulting supernatant was filtered in MultiScreen HV 96‐well filtration plates at 1500× g for 2 min (Durapore®, 0.45 μm, Millipore, USA) to remove debris. A Protein A HPLC method was used to measure the monoclonal antibody titer using a 2695/2996 HPLC system (WATERS Corporation, USA) with a protein A cartridge (POROS® A20 column, 2.1 mmD × 30 mmH, Thermo Fisher Scientific, Part# 2–1001‐00). The column was equilibrated with phosphate buffered saline solution without calcium and magnesium (Cat. No. SH30028.03, Cytiva, USA) and then samples were loaded at 2 mL/min. The column was washed with 1 mL, 10 column volumes, to remove unbound species and cell culture media components. The attached antibodies were eluted using 0.15 M NaCl solution at pH 2.0 for 1 min. Detection of the antibody product is done by UV at 280 nm. The standard error of the measurement using this method was typically below 10%. Cell specific mAb production (qP, pg/cell/day) is calculated from (Equation 9) where P (pg/mL) is the product volumetric concentration measured at two timepoints. The space time yield (STY, [mg/L/day]) is calculated according to (Equation 10) where the product concentration is given in mg/L and the time is given in days.

(9) $$\mathrm{qP}=\frac{P_{2}-P_{1}}{∆\text{IVCC}}$$

(10) $$\mathrm{STY}=\frac{{\left[\text{Product}\right]}_{\text{final}}}{{\text{Time}}_{\text{final}}}$$

## 3 RESULTS AND DISCUSSION

### 3.1 Effect of feeding strategy

**FIGURE 2:**
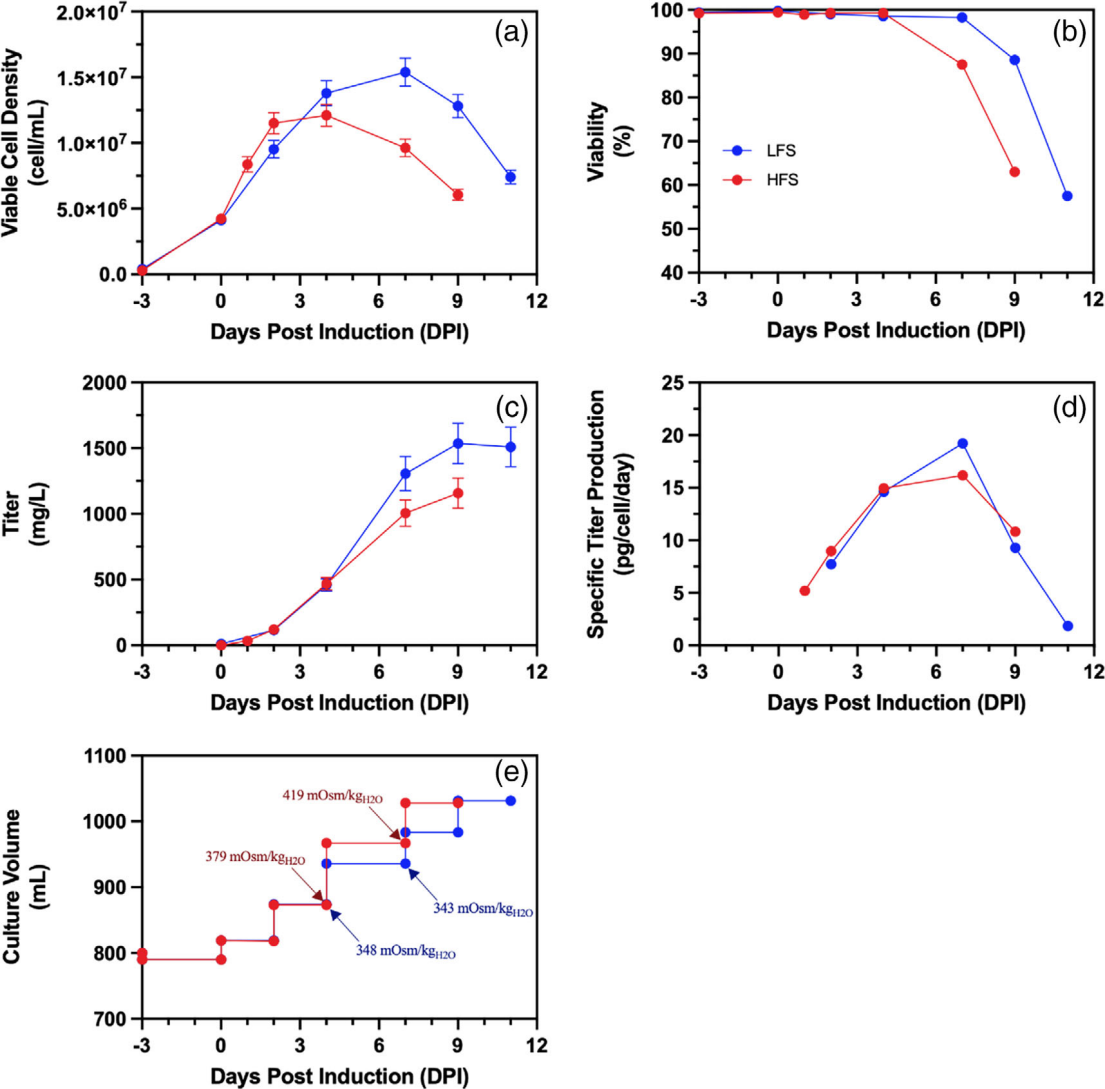
The effect of bolus feeding strategy on the (a) viable cell density (cell/mL), (b) cell viability (%), (c) volumetric titer (mg/L), (d) cell specific titer (pg/cell/day), and (e) cumulative cell culture volume (mL) of 1‐L benchtop bioreactor cell cultures. Low total volume feeding strategy (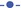); High total volume feeding strategy (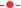). Cell cultures are presented as single runs. Error bars represent measurement errors for viable cell density (a) and volumetric product titers (c).

Cell cultures following low (LFS) and high (HFS) feeding strategies had doubling times of 21.5 and 18.8 h, respectively, during the growth phase between −3 and 0 DPI. The difference in doubling times is within the observed range of variability during the seed train while maintaining cells in the exponential growth phase. This resulted in a comparable viable cell density (VCD) and cell viability (Figure 2a,b) at the end of the growth phase (0 DPI). The comparability of both cell cultures extends to metabolite (glucose, lactate, and ammonia) concentrations, as well as cell‐specific consumption and production. The growth phase exhibits the highest lactate production throughout the cell culture, with 2.4 and 2.8 pmol/cell/day of lactate produced while 0.87 and 1.2 pmol/cell/day of glucose are consumed for the LFS and HFS cell cultures, respectively (Supplemental Figure 1). This is consistent with literature where rapid cell growth of CHO cells is associated with high glycolytic activity and thus lactate production.^30^

At the beginning of the production phase, between 0 and 4 DPI, the difference in VCD for both feeding strategies is at most 17%. During this period, specific glucose consumption stays consistent at just below 1 pmol/cell/day for the LFS culture, while the specific glucose consumption for the HFS culture jumps rapidly to 3 pmol/cell/day at 1 DPI before decreasing to near zero at 4 DPI (Supplemental Figure 1B). Lactate levels reach a peak during this period, and cell metabolism enters lactate consumption mode for both feeding conditions (Supplemental Figure 1C). After induction, cell specific PLVZM mAb production increases linearly until 4 DPI, reaching 14.6 and 14.9 pg/cell/day for the LFS and HFS, respectively. LFS and HFS cell cultures attain 459 and 469 mg/L, respectively, at 4 DPI, with comparable specific productivities and VCDs. Differences in VCD and glucose consumption are presumed to be due to the difference in glucose supplementation, although this has no significant impact on titer.

Between 4 and 9 DPI, the HFS culture gradually loses cell viability, while the LFS culture maintains cell viability above 98% until 7 DPI, after which it decreases until 11 DPI (Figure 2b). The early drop in viability of the HFS culture occurred following culture supplementation at 4 DPI (12% of the initial volume in feed, compared to 7.5% for the LFS culture). With all else being the same between these two cultures, the crash in cell viability was seemingly caused by a larger and rapid increase in osmolality from the addition of a concentrated bolus feeding solution (feed's osmolality is in the range of 780–930 mOsm/kg). As a result of this higher feed volume between 4 and 7 DPI, the osmolality of the culture media increased from 379 to 419 mOsm/kg, whereas in the lower feed addition culture (LFS), osmolality was maintained approximately constant at 348 and 343 mOsm/kg (Figure 2e). The osmolality of the HFS culture, at 419 mOsm/kg at 7 DPI, is close to the critical osmolality of 450 mOsm/kg for CHO cell cultures, which has been reported to negatively impact cell viability and VCD.^17^, ^19^, ^31^, ^32^, ^33^ It has previously been noted in the literature that a reduction in bolus feeding amount allowed for lower osmolality and increased product titers.^34^ In a separate study done by our research group, large bolus feeding of comparable proportions using similar operating parameters in benchtop bioreactors resulted in early cell death when producing the SARS‐CoV‐2 recombinant spike protein in a CHO stable cell pool.^17^ Since cell death probably resulting from a comparable osmolality increase was also observed in a CHO cell clone producing a different recombinant protein, it is reasonable to argue that overfeeding of this magnitude is detrimental to cell viability. Different strategies have been suggested in the literature to mitigate the adverse effects of high bolus feeding. The CHO cells can be adapted to hyperosmotic conditions over several cell passages to better tolerate high osmotic environments.^35^ The feeding technique could also be adapted to feed the same volume continuously, which allows for the maintenance of low nutrient concentrations and a manageable osmotic pressure, allowing for a longer preservation of cell viability.^17^ Overfeeding may have other negative implications on cell culture performance as high concentrations of certain feed nutrients may decrease cell growth, productivity, and protein quality.^36^ Specifically, TCA cycle intermediates (malate and citrate) have been observed to accumulate after feeding, which has been linked to growth limitations.^11^, ^36^

Due to the decrease in viability, the VCD of the HFS culture is negatively impacted with respect to the LFS culture. This early reduction in viability with greater feeding is reflected in the cell‐specific productivity, which is constant at ~15 pg/cell/day from 4 to 7 DPI, after which it decreases as cell viability further descends. With lower feeding, cell‐specific productivity continues to increase until ~19 pg/cell/day before sharply decreasing during cell death, reaching near zero when cell viability is below 70% (Figure 2d). The early loss in cell viability attributed to the bolus increase in osmolality led to a lower volumetric titer (Figure 2c). Here, cell‐specific productivity dropped because of cell death presumably from osmotic stress caused by the increased feed amount. In the literature, the positive effects of osmotic stress on cell‐specific productivity toward the end of production have been well documented.^30^, ^31^ Specifically, a high osmotic pressure induces an increase in cell size,^32^ which in turn is known to boost cell‐specific productivity.^33^, ^34^ Furthermore, a higher volume feeding regimen will lead to a higher final production volume, which could dilute the product concentration. As a result, even if a higher product concentration is obtained with a low feeding regimen, it could still lead to a lower total product yield. However, this is not the case here; the low‐volume feeding regimen bioreactor yielded 1511 mg at a final volume of 974 mL, while the high‐volume feeding regimen produced 1190 mg at a final volume of 1018 mL.

### 3.2 Effect of impeller number and aeration strategy

**FIGURE 3:**
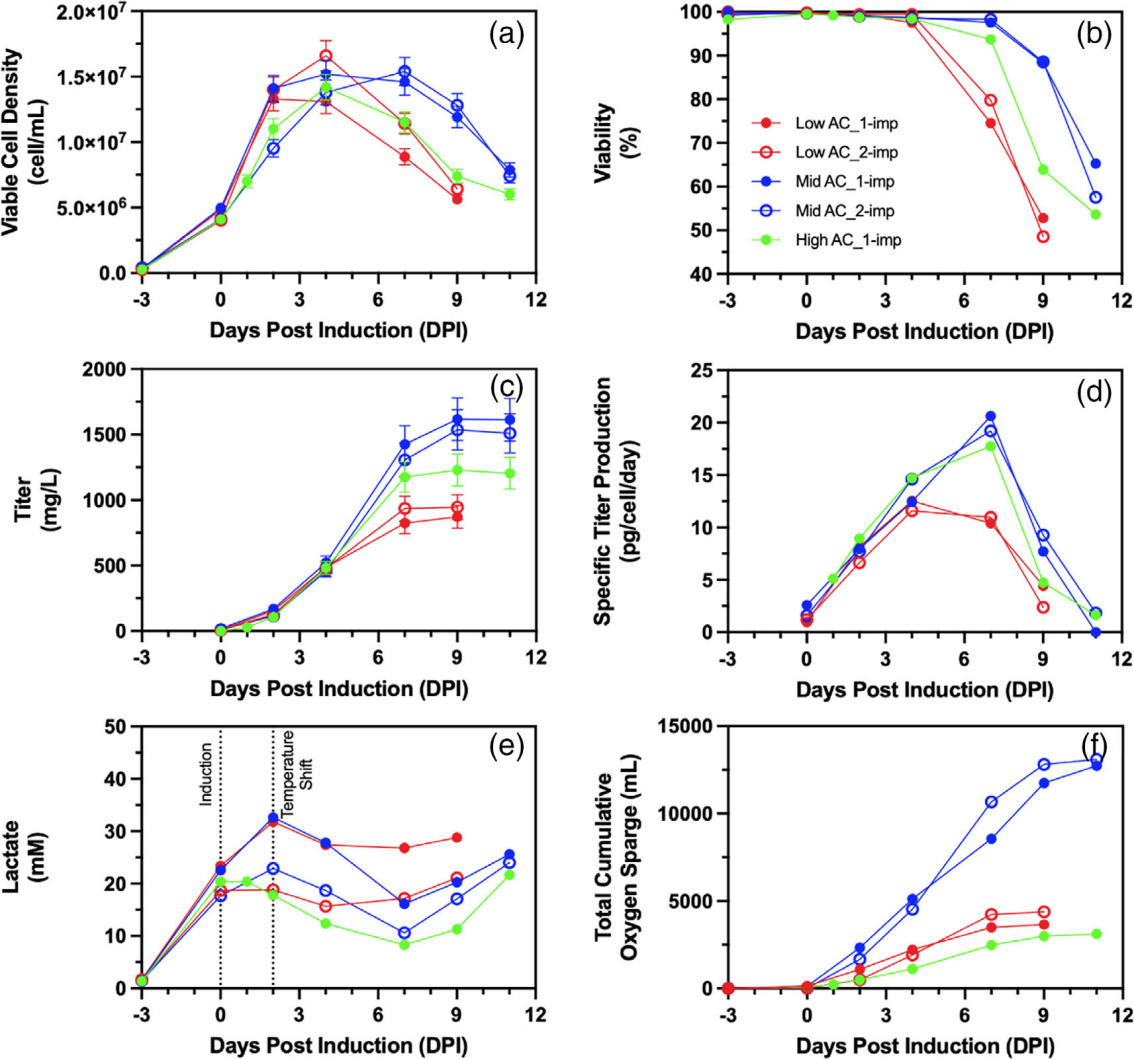
Effect of impeller number and aeration strategy on (a) viable cell density (cell/mL), (b) cell viability (%), (c) volumetric titer (mg/L), (d) cell specific titer (pg/cell/day), (e) lactate concentration (mM), and f) total cumulative oxygen sparged from air and pure oxygen (mL) for low (1 mL/min, red), mid (2 mL/min, blue), and high (12.3 mL/min, green) air caps; 1‐impeller configuration (full dots) and 2‐impeller configuration (empty dots). Cell cultures are presented as single runs. Error bars represent measurement errors for viable cell density (a) and volumetric titers (c).

Oxygen control of bioreactor cell cultures was achieved through cascade aeration, whereby air is sparged until a pre‐set maximal value. When this air cap (AC) flow rate is reached, pure oxygen is supplied on demand to maintain dissolved oxygen setpoints. As no air or oxygen had been sparged during the growth phase, from −3 to 0 DPI (Figure 3f and Supplemental Figure 2), all studied air cap conditions have comparable cell growth (Figure 3a), and differences in cell metabolism prior to induction are expected to be due to cell culture variability. From 0 to 7 DPI, which is the period during which most protein production occurs (Figure 3c,d), cell‐specific glucose consumption is comparable among all culture conditions (data not shown). For all conditions, lactate levels continue to increase until 2 DPI, when a temperature shift occurs, as the cells are still in the exponential growth phase (Figure 3e).^35^ After the temperature shift, the cell metabolism changes to lactate consumption. In the case of the 1 mL/min low air cap condition, the maintained lactate levels from 4 DPI onwards may be due to early cell death.

Gas sparging during the exponential growth phase has been shown to significantly retard cell growth as the cells adapt to the high shear stress environment.^36^ Thus, the aeration cascade gassing strategy may allow for a greater cell growth by minimizing gas sparging (Figure 3f). Headspace air aeration is sufficient to support cell growth from −3 DPI to 0 DPI. Among the three different levels of air cap in this study (low, mid, high), the mid air cap condition with 1‐impeller yielded a maximum volumetric product titer of 1617 mg/L at 9 DPI (Figure 3c). Meanwhile, in the high AC condition, the maximum volumetric product titer was 1228 mg/L at 9 DPI due to an earlier cell death and lower VCD compared to the mid AC condition. Using a higher air cap required a greater total volume of gas sparging, which is expected, as a larger proportion of the oxygen supplied to the cells comes from air. At 11 dPI, a total of 34.9 L of gas (air, oxygen, and carbon dioxide) was sparged in the mid AC condition compared to 76.6 L of total gas sparged in the high AC condition (Supplemental Figure 2). This larger total volume of sparged gas applied a greater shear stress on the cells, as it is well known that the bursting of bubbles at the surface of the liquid is one of the most significant contributors to shear stress.^37^ High sparging rates also improve CO_2_ stripping; sustained ultra‐low pCO_2_ levels have been shown to reduce VCD and cell viability.^38^ When using the lower air cap gassing condition with 1‐impeller configuration, a maximum titer of 872 mg/L was obtained at 9 DPI. The low air cap condition had the earliest observed cell death with 74.5% cell viability at 7 DPI (Figure 3b). This early cell death is reflected in the specific titer production, which is lower than in the other two conditions at 7 DPI (Figure 3d). The low AC condition had the lowest total gas sparging of 12.1 L at 11 DPI. A reduction in gas sparging, in turn, decreases CO_2_ stripping rates. Elevated pCO_2_ levels have been shown to reduce cell growth rate and productivity.^19^ In addition to this, low pCO_2_ is desirable as it has been correlated with improved mAb galactosylation.^38^ Although pCO_2_ was not measured here, it can be assumed there is no accumulation, as this is mainly an issue at larger scales due to longer gas residence times leading to bubbles becoming saturated with CO_2_ before reaching the liquid surface.^6^, ^37^, ^39^

The cell growth, viability, and productivity of 1‐L bioreactor CHO cell cultures are comparable with 1‐ and 2‐impeller bioreactor configurations (Figure 3). In a 2‐impeller bioreactor system, with equivalent volumetric power input to a 1‐impeller system, the impeller tip speed will be slower, allowing a lower local maximum shear rate by spreading out the mixing zones over two impellers.^40^ In 1‐ and 2‐impeller systems, cells experience the same shear stress from bursting gas bubbles at the surface. Therefore, dual‐impeller systems are expected to apply lower overall shear stress to cells than single‐impeller systems. A lower maximum lactate concentration is observed in the 2‐impeller system when compared with the 1‐impeller system for both the low AC and mid AC conditions (Figure 3e). This is in accordance with the literature, as high shear stress environments are known to cause greater lactate production and delayed lactate consumption phases.^17^, ^41^ Additionally, multi‐impeller systems are known to have more efficient gas distribution, higher gas residence times, and increased gas hold‐up.^27^ However, in this case, shear stress on cells does not appear to be an issue, as both 1‐ and 2‐impeller configuration cell cultures are comparable.

### 3.3 Scale‐up from 1‐L to 10‐L bioreactor

**FIGURE 4:**
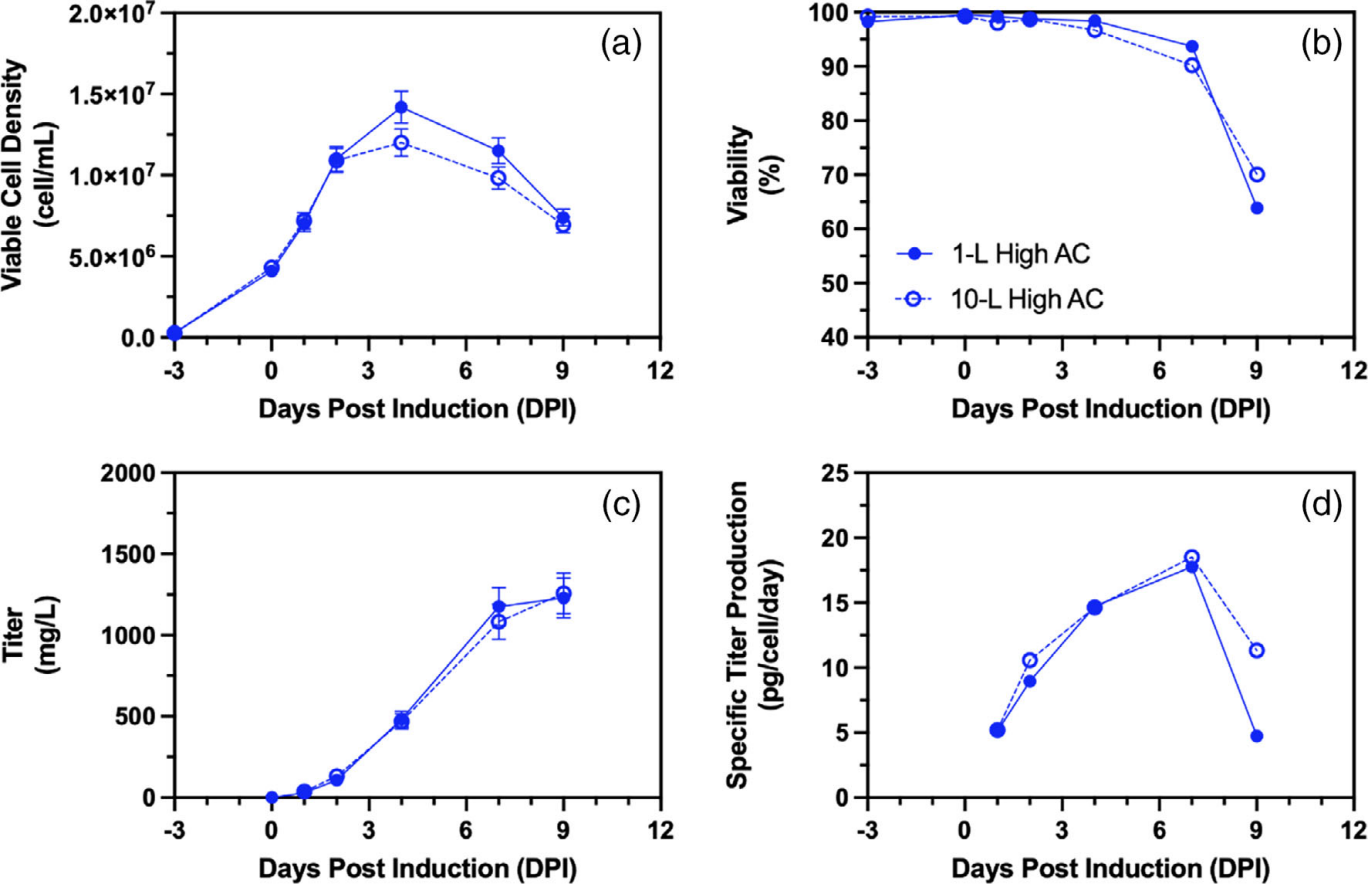
Scale‐up of 1‐L benchtop bioreactor to 10‐L bioreactor using constant P/V (22 from −3 to 2DPI then 29 W/m^3^) and vvm (0.015) as criterion and their measured (a) viable cell density (cell/mL), (b) cell viability (%), (c) volumetric titer (mg/L), and (d) cell specific titer (pg/cell/day) obtained in 1‐L benchtop bioreactor with high AC aeration (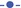) and 10‐L benchtop bioreactor with high AC aeration (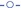). Cell cultures are presented as single runs. Error bars represent measurement errors for viable cell density (a) and volumetric titers (c).

Although the 1‐L mid AC aeration condition was observed as the optimal condition for volumetric productivity (Figure 3), the 1‐L High AC cell culture was selected for process scale‐up to 10‐L. This decision was based on the need for higher vvm typically required in large‐scale bioreactors to prevent CO_2_ accumulation as well as physical gas flowrate limitations of the 10‐L bioreactor. The 10‐L bioreactor was equipped with thermal mass flow controllers (TMFCs) for both pure oxygen and air. However, the maximum oxygen flow capacity of the TMFC was insufficient to maintain dissolved oxygen (DO) levels at or above the set point when using an air cap vvm similar to the mid AC condition in previous cultures performed by our research group (data not shown), necessitating the use of the High AC strategy. Scale‐up from 1‐L to 10‐L was performed using a constant volumetric power input (P/V) as a primary scale‐up criterion as this was shown to be the most reliable approach for small differences in operating vessel volumes.^8^ Volume‐normalized gas flow rate (vvm) was selected as a criterion for gas sparging. The combination of constant P/V and vvm across scales has been shown to result in comparable VCD, viability, and product titer between 3‐L, 500‐L, and 2000‐L bioreactors.^42^ The constant vvm allows for sufficient CO_2_ stripping and oxygen transfer while the constant P/V ensures proper mixing. Due to maximum gas flowrate limitations in the 10‐L system, the maximum vvm was increased from 0.006 to 0.015 to ensure DO setpoint levels could be maintained. This scale‐up strategy led to comparable cell growth and viability (Figure 4a,b), volumetric and cell‐specific productivities (Figure 4c,d) between 1‐L and 10‐L productions.

### 3.4 Process transfer to a different product

**FIGURE 5:**
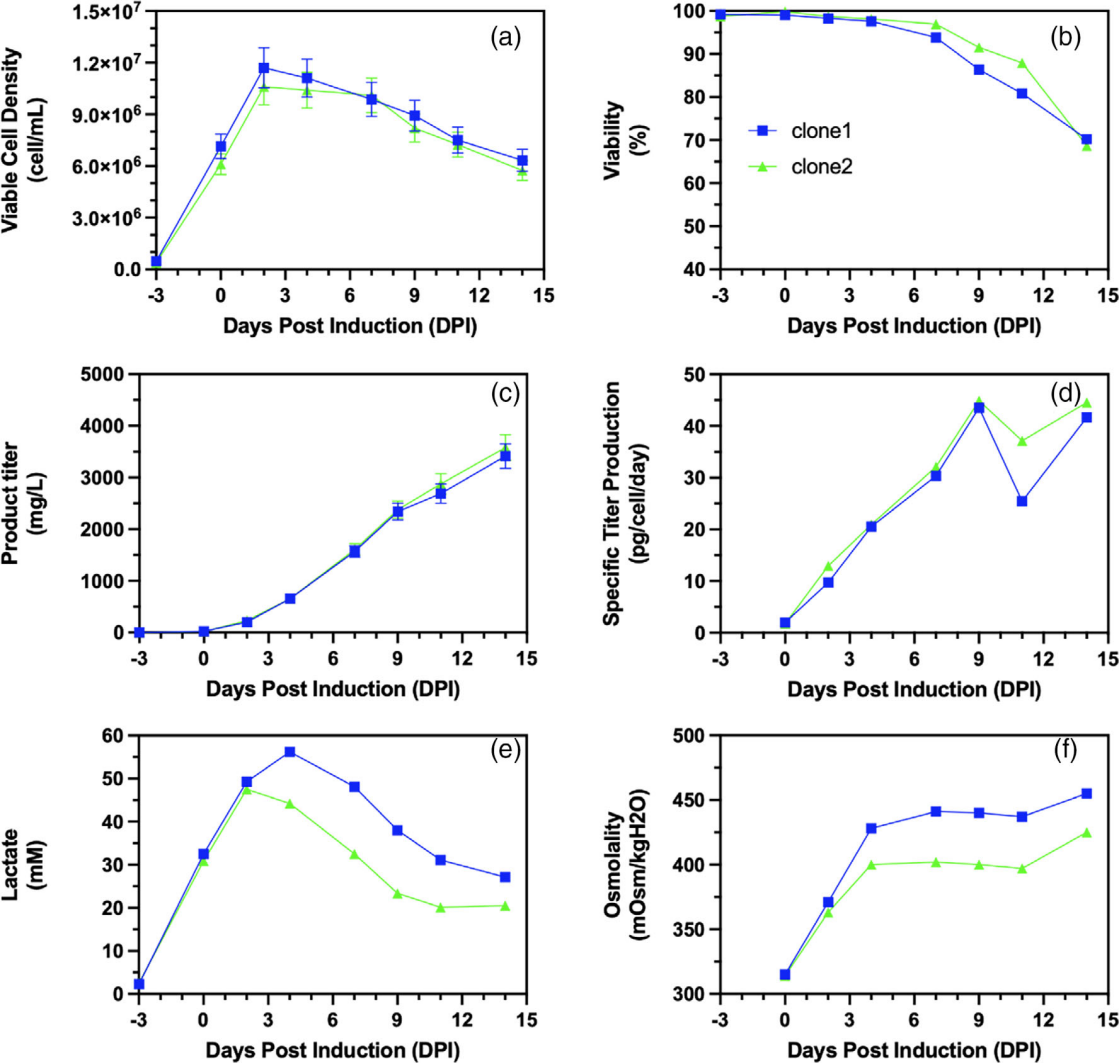
Benchtop 1‐L bioreactor cell culture performance of clone 1 (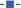 ), and clone 2 (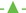) for the production of Product B operating at mid AC (0.0031vvm) with single impeller and LFS (37.5% of initial volume feed volume) (a) viable cell density (cell/mL), (b) cell viability (%), (c) volumetric titer (mg/L), (d) cell specific titer (pg/cell/day), (e) lactate concentration (mM), and (f) osmolality (mOsm/kg_H2O_). Cell cultures are presented as single runs. Error bars represent measurement errors for viable cell density (a) and volumetric titers (c).

The same culture strategy was assessed with two clones expressing another antibody (Product B) in benchtop bioreactors. Both clones were observed to achieve comparable VCD, viability, volumetric, and cell‐specific product profiles (Figure 5a–d). However, in clone 2, the metabolic switch to lactate consumption occurs earlier (after 2 DPI) than in clone 1 (after 4 DPI) (Figure 5e). The production of lactate is associated with a reduction of pH as lactate is excreted from cells through a monocarboxylate transporter (MCT), which also excretes an H^+^ ion as it is a co‐transporter.^43^ In pH‐controlled cell cultures, base is added when pH reaches the lower limits of the deadband. This addition of base, in turn, increases the osmolality of the culture media. This explains the greater osmolality observed with clone 1 when compared to clone 2 (Figure 5f). The switch in lactate metabolism from production to consumption is driven by many different factors, such as low glucose levels or depletion, glutamine depletion, high lactate concentration, high H^+^ concentration (low pH), and the transition of cell growth to the stationary phase.^44^ Another viable explanation for the lactate metabolic switch is that it is controlled by redox balancing.^44^ The sole source of lactate production is from the reduction of pyruvate catalyzed by lactate dehydrogenase (LDH). This conversion also oxidizes NADH to NAD^+^. Thus, when NADH levels rise, such as during high glycolytic activity, rates of conversion of pyruvate to lactate increase. Meanwhile, it was shown that lactate excretion and consumption rates were solely determined by the concentration gradients of both lactate and H+ ions.^45^ Although no difference in cell culture productivity was observed as a result of the difference in lactate and osmolality, clone 2 was deemed a better performer as high lactate and osmolality levels are typically associated with shorter culture duration and lower productivities. Thus, clone 2 was used for investigating the effect of hydrodynamic conditions on productivity in the following section.

**FIGURE 6:**
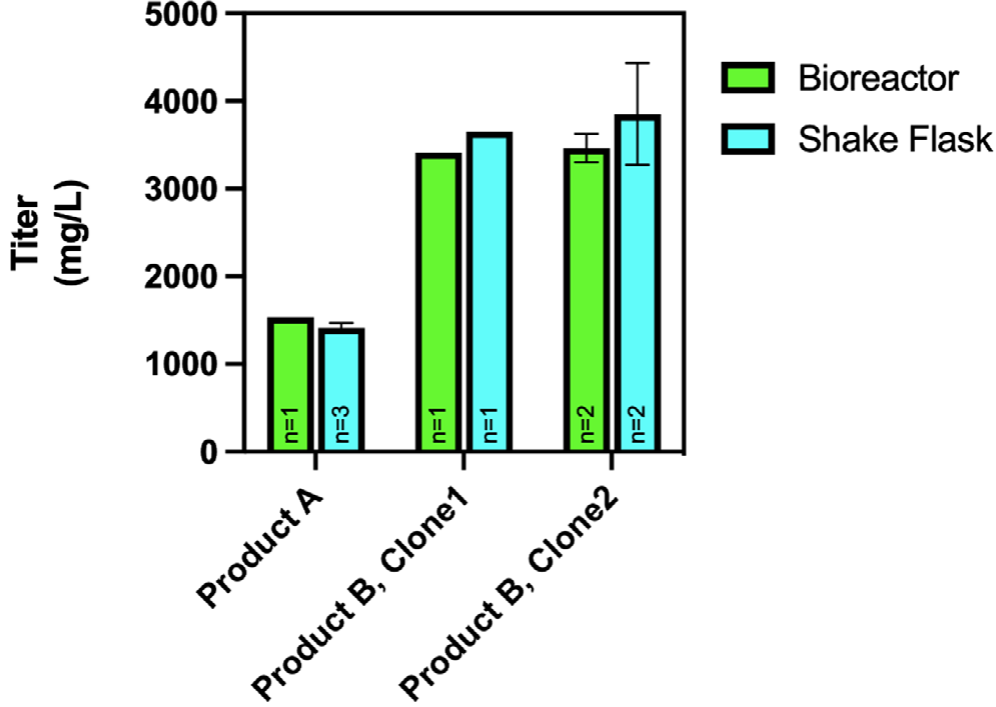
Comparison of final volumetric product titer (mg/L) of product A, product B from clone 1, and product B from clone 2 in 1‐L bioreactor (green) and shake flask (blue). Bioreactors were operating at mid AC (0.0031vvm) with single impeller and LFS (37.5% of initial volume feed volume). Error bars of biological replicates represent standard deviations.

It is worth mentioning that all bioreactor cultures (Product A, Product B clone 1, and Product B clone 2) produced 90%–109% of their respective external control shake flasks (125 mL baffled flask, 200 RPM, 25 mm orbit) titer (Figure 6), showing the linearity of scale‐up. For a given product, the average cell‐specific productivity, VCD, and titer profiles in shake flask cultures were similar to the ones in bioreactors (shown in Figures 4 and 5). The consistent titers at 9 DPI (Product A) and 14 DPI (Product B) between the flask and bioreactor indicate that the process developed by optimizing feeding regimens, mixing, and gassing strategies is robust, and that comparable parameters are suitable across different mAb products and cell lines. It is also worth noting that the biological replicate cultures performed in bioreactors (and shake flasks) yielded comparable titers within the variance of the measurement technique.

### 3.5 Effect of culture hydrodynamic conditions on productivity

In a fed‐batch bioreactor, as the culture progresses, the working volume increases, which changes variables such as the volumetric power input (P/V) and volumetric gas flowrate (vvm). The P/V is an important engineering parameter in bioreactor process design due to its implications for mixing and distribution of nutrients, gradients of pH, oxygen and carbon dioxide transfer, and shear stress considerations. As for vvm, we have shown in this study the importance of its optimization for improvements in cell longevity and productivity. To assess the impact of both P/V and vvm on cell growth, viability, and productivity, the process previously developed with LFS, mid AC aeration, and 1‐impeller to produce Product B in clone 2 was used as a standard process. However, maintaining P/V and vvm of AC constant throughout the cell culture, by adjusting mixing speed and gas flowrate at AC, has impacts on other crucial engineering variables such as the impeller tip speed, superficial gas velocity, and gas entrance velocity. To further remove the impact of these parameters, an additional culture was performed where the culture volume was kept constant by removing an amount of culture broth equal to the feed volume after each feeding.

**FIGURE 7:**
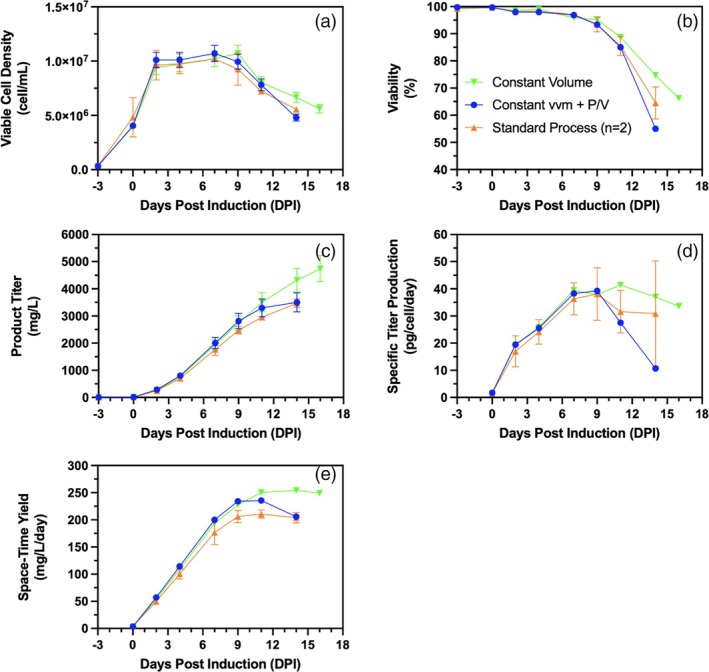
Effect of constant volumetric gas flowrate (vvm) at air cap, constant volumetric power input (P/V), and constant volume on (a) viable cell density (cell/mL), (b) cell viability (%), (c) volumetric titer (mg/L), (d) cell specific titer (pg/cell/day), (e) space–time yield (mg/L/day); Constant volume cell cultures (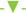 ), constant air cap vvm and P/V cell cultures (no volume adjustments) (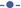 ), and the standard process with middle AC, constant mixing speed, with variable volume from bolus feeding (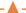 ). Bioreactors were operating at an initial mid AC (0.0031vvm) with single impeller and LFS (37.5% of initial volume feed volume). Error bars of duplicates represent standard deviation while error bar of single runs represent measurement errors for viable cell density (a) and volumetric titers (c).

Maintaining consistent hydrodynamic conditions, either by keeping the culture volume constant or by adjusting mixing speed and air cap flow rate to maintain a constant P/V and air cap volumetric gas flow rate (vvm) throughout cell culture, led to an improvement in space–time yield (STY) up to 11 days post‐induction (DPI). This suggests that the uniformity of these engineering variables plays a crucial role in enhancing overall productivity. Furthermore, maintaining a constant culture volume helped sustain a high STY up to 16 DPI (Figure 7e). This is potentially because increasing P/V and vvm has impacts on shear stress applied to cells at the impeller tip and liquid surface, respectively. Mixing times in small‐volume bioreactors are generally adequate in all situations when P/V is between 10 and 80 W/m^3^. However, constant volumes allow for maintaining a lower mixing speed while keeping P/V constant. To maintain P/V constant in a fed‐batch cell culture, the mixing speed of the impeller must be increased at each bolus feeding point. This will naturally increase the impeller tip speed, thereby increasing the shear stress applied to cells. Increasing vvm will cause a higher total gas sparging rate, potentially leading to increased cell damage over the course of the cell culture. The constant volume culture yielded a more favorable environment for cell growth and protein production, which underscores the importance of carefully balancing engineering parameters to optimize cell culture performance.

The volumetric power input and gas flow rates not only have an impact on shear stress applied to cells but also on gas transfer. The volumetric gas transfer coefficient, k_L_a, is used to express the rate of transfer from the gas to the liquid phase. In the case of bioreactors, the gas phase represents the sparged bubbles and the liquid phase is the bulk media. As shown in (Equation 11), k_L_a is a function of mixing (P/V) and aeration (v_s_), where v_s_ is the superficial gas velocity and K, α, and 𝛽 are empirically determined constants.^46^

(11) $$k_{L}a=K\times {\left(\frac{P}{V}\right)}^{\alpha }\times {v_{s}}^{\beta }$$

In the three process operating conditions presented (Figure 7), cell cultures begin with the same volume, agitation rate, and gassing such that the gas transfer rates are the same. The k_L_a at the beginning of the culture, which was previously measured using the cell‐free static gassing out method, was of 14 h^−1^ (data not shown). In the case of the standard process, the culture volume increases with time due to feed additions, and the agitation speed stays constant over time. This decreased the P/V by 19%, which reduced the k_L_a to 12 h^−1^ by the end of the cell culture. However, in the case of the constant P/V and vvm and the constant volume condition, the mixing and aeration rates are adjusted such that k_L_a is maintained constant throughout the cell culture. The same applies to the constant volume cell culture where mixing speed and gas flowrate at the air cap are fixed, theoretically enabling a constant k_L_a. An unchanged P/V and vvm strategy shows an improvement in titer but requires an increase in agitation speed, while a constant volume shows a further increase in titer without the requirement for an increase in mixing speed.

## 4 CONCLUSION

The objective of this study was to optimize CHO cell cultures in benchtop bioreactors for parameters that are particularly important during scale‐up: aeration and mixing. Before aeration and mixing could be optimized, a feeding regimen needed to be selected. It was shown that cell cultures with a high bolus feeding regimen generating spikes in osmolality exhibited an early drop in cell viability. A lower feeding regimen enabled 25% more final volumetric titer concomitantly with a significantly improved cell growth. Different strategies could be employed to avoid the adverse effects of an increase in osmolality. Namely, adapting the clone to a high osmolality environment by gradually increasing the osmolality of the culture media, or similarly, feeding the same quantity continuously rather than in bolus.^17^ The importance of optimizing aeration strategies was shown by utilizing different air caps with cascade aeration. As the air cap was increased, a larger total volume of gas was sparged throughout the cell culture. It is suspected that the low VCD and faster decline in cell viability associated with the higher air cap were due to the prolonged shear stress caused by higher gas sparge rates. To assess process scalability, the 1‐L process was transferred to a 10‐L vessel using a constant P/V and vvm strategy. A comparable cell culture performance, in terms of VCD, viability, and productivity, was observed. For scale‐up across larger volumes, constant P/V and vvm have been shown to be successful when the gas flow rate is sufficient to ensure efficient CO_2_ stripping.^8^ In cases where these parameters do not produce an environment with sufficient gas transfer rates, constant P/V could be paired with constant k_L_a at the air cap to ensure that DO is maintained at the setpoint. The optimized 1‐L process was then transferred to two different cell lines expressing a different product. A linearity showing 90%–109% of shake flask titer was obtained demonstrating the robustness of the developed process. Studying the effect of mixing and aeration on cell culture productivity revealed the delicate interplay between sufficient mixing, efficient gas transfer, and the stress applied to cells.

## AUTHOR CONTRIBUTIONS

**Lucas Lemire**: Conceptualization; methodology; data curation; investigation; validation; writing – original draft. **Sebastian‐Juan Reyes**: Formal analysis; writing – review and editing. **Yves Durocher**: Supervision; resources; formal analysis; writing – review and editing. **Robert Voyer:** Resources; writing – review and editing. **Olivier Henry** and **Phuong Lan Pham**: Supervision; formal analysis; validation; funding acquisition; writing – review and editing; resources; project administration.

## CONFLICT OF INTEREST STATEMENT

The authors declare no conflicts of interest.

## Supporting information


**Supplemental Figure 1:** The effect of feeding strategy on the (A) residual glucose, (B) specific glucose consumption, (C) measured lactate, (D) specific lactate production (E) ammonia concentration, and (F) specific ammonia production of 1‐L benchtop bioreactor cell cultures. Low feeding strategy (−●‐ LFS); High feeding strategy (−●‐ HFS).
**Supplemental Figure 2:** Online sparging data (A) air, (B) pure oxygen, and (C) carbon dioxide for low air cap with 1‐impeller (red), mid air cap with 1‐impeller (blue), and high air cap with 1‐impeller (green) operating parameters.

## Data Availability

The data that support the findings of this study are available from the corresponding author upon reasonable request.
