# Supplementary material for: Scale‐up of a monoclonal antibody CHO fed‐batch production in stirred tank bioreactors: Effect of hydrodynamic conditions and feeding regimen
Source: Biotechnol Prog. 2025 Sep 29;42(1):e70073. doi: 10.1002/btpr.70073 (PMC12908111; doi:10.1002/btpr.70073)
Supplement: Supplementary file 1 — Supplemental Figure 1: The effect of feeding strategy on the (A) residual glucose, (B) specific glucose consumption, (C) measured lactate, (D) specific lactate production (E) ammonia concentration, and (F) specific ammonia production of 1‐L benchtop bioreactor cell cultures. Low feeding strategy (−●‐ LFS); High feeding strategy (−●‐ HFS). Supplemental Figure 2: Online sparging data (A) air, (B) pure oxygen, and (C) carbon dioxide for low air cap with 1‐impeller (red), mid air cap with 1‐impeller (blue), and high air cap with 1‐impeller (green) operating parameters. [file BTPR-42-e70073-s001.docx]

# SUPPLEMENTARY DATA


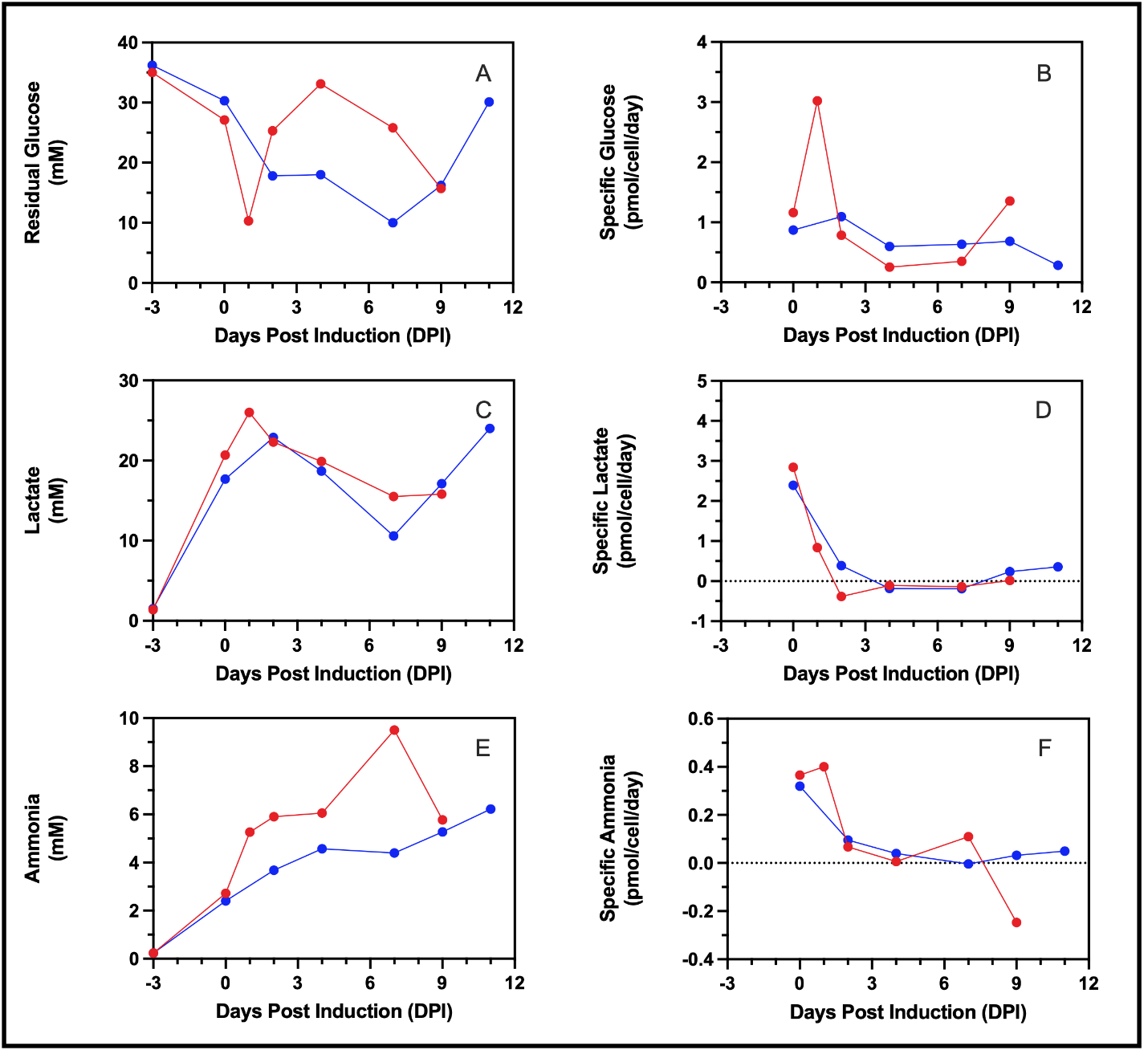


Supplemental Figure 1: The effect of feeding strategy on the A) residual glucose, B) specific glucose consumption, C) measured lactate, D) specific lactate production E) ammonia concentration, and F) specific ammonia production of 1-L benchtop bioreactor cell cultures. Low feeding strategy (-●- LFS); High feeding strategy (-●- HFS).


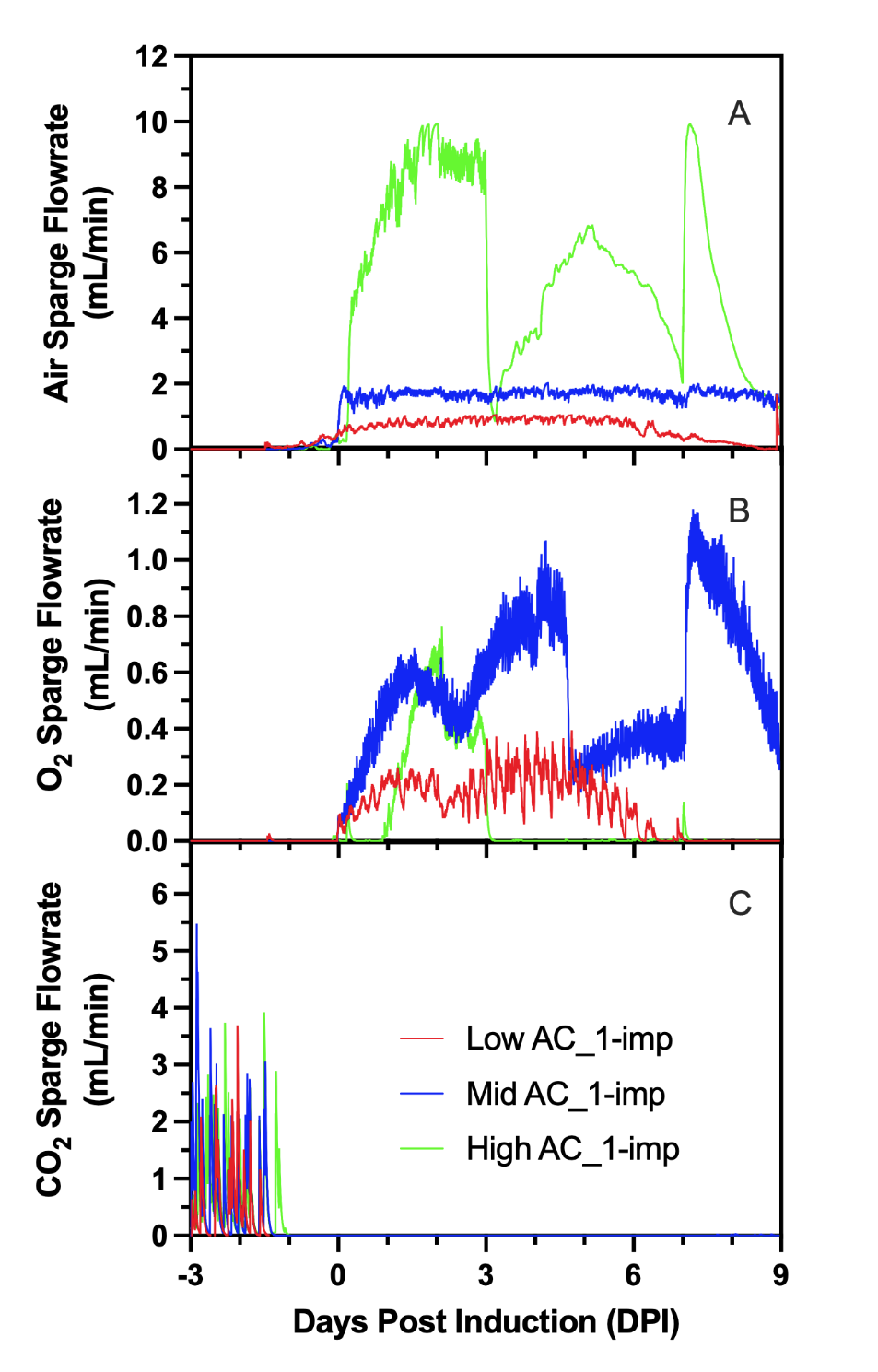


Supplemental Figure 2: Online sparging data A) air, B) pure oxygen, and C) carbon dioxide for low air cap with 1-impeller (red), mid air cap with 1-impeller (blue), and high air cap with 1-impeller (green) operating parameters.
